# Levels of 25-hydroxyvitamin D_3_ in individuals with primary subjective tinnitus and their associations with tinnitus occurrence and severity

**DOI:** 10.3389/fneur.2025.1751366

**Published:** 2026-01-12

**Authors:** András Molnár, Panayiota Mavrogeni, Aphrodite Mavrogenis, Stefani Maihoub

**Affiliations:** 1 Opera Clinic, Protone Audio Kft., Budapest, Hungary; 2 Faculty of Health Sciences Doctoral School, University of Pécs, Pécs, Hungary; 3 Bajcsy–Zsilinszky Hospital, Budapest, Hungary; 4 Maihoub ENT Clinic, Limassol, Cyprus

**Keywords:** 25-hydroxyvitamin D_3_ levels, chronic subjective tinnitus, tinnitus, tinnitus intensity, tinnitus severity

## Abstract

**Objective:**

This study aimed to analyze vitamin D_3_ levels in both a tinnitus group and a control group.

**Materials and methods:**

A total of 350 patients with primary subjective tinnitus and 347 participants serving as a control group were examined. All patients underwent an otorhinolaryngological examination, audiological testing, and laboratory testing, including measurements of 25-hydroxyvitamin D_3_ levels.

**Results:**

The prevalence of low 25-hydroxyvitamin D_3_ levels was significantly (*p* < 0.00001) higher in the tinnitus group (53.2%) compared to the control group (31.7%). Furthermore, when comparing the specific levels of 25-hydroxyvitamin D_3_ between the tinnitus and control groups, a statistically significant difference was observed (*p* < 0.00001), with lower levels found in the tinnitus group. Additionally, according to a logistic regression model, lower levels of 25-hydroxyvitamin D_3_ significantly predicted the occurrence of tinnitus (*p* = 0.000; OR: 0.132, 95% CI = 0.066–0.264). Additionally, it was found to be associated with moderately severe and severe tinnitus (*p* = 0.021; OR: 1.800, 95% CI = 0.806–4.022) and the development of chronic tinnitus (*p* = 0.001; OR: 0.717, 95% CI = 0.384–1.340). Moreover, ROC analysis indicated that lower levels of 25-hydroxyvitamin D_3_ are significant predictors of tinnitus (*p* = 0.000, 95% CI = 0.702–0.815), showing a sensitivity of 75.8%.

**Conclusion:**

The notably lower levels of 25-hydroxyvitamin D_3_ suggest its potential role in the development of tinnitus. Additionally, proper supplementation of vitamin D_3_ could lead to new therapeutic approaches for managing tinnitus.

## 1 Introduction

Tinnitus is a sound that originates without an external source, primarily experienced subjectively by the individuals affected. Tinnitus affects approximately 15% of the population (1). Tinnitus that is not managed appropriately greatly affects quality of life and leads to psychiatric symptoms, particularly depression and anxiety (2). Tinnitus can be classified into two main categories: primary and secondary cases. Primary cases include idiopathic tinnitus and those associated with sensorineural hearing loss. In contrast, secondary cases involve various conditions such as inflammation of the outer and middle ear, earwax buildup, otosclerosis, and Ménière’s disease (3, 4). The most common type of tinnitus is chronic idiopathic tinnitus, which means that, in many instances, no specific cause can be identified (5). However, it is important to note that potential systemic causes of tinnitus are often overlooked in many cases. A significant shift in recognizing tinnitus as a critical warning signal is essential.

Vitamin D is a fat-soluble secosteroid that exists in two forms: D_2_, found in plants and fungi, and D_3_, which is synthesized in humans from 7-dehydrocholesterol in the skin when exposed to UV light (6). Vitamin D_3_ is then transported to the liver, where it is converted by the enzyme CYP2R1 into 25-hydroxyvitamin D_3_ [25(OH)D_3_], the primary circulating form of vitamin D (7). Finally, in the kidneys, the CYP27B1 enzyme converts it to its active form, 1,25-dihydroxyvitamin D_3_ [1,25(OH)2D_3_] (8). In addition to its well-established importance in musculoskeletal functions, 25-hydroxyvitamin D_3_ [25(OH)D_3_], plays vital roles, e.g., in brain functions, the immune system, cardio-vascular health, and cancer prevention (9). Vitamin D receptors (VDRs) are found in various tissues throughout the body, including the brain, immune cells, prostate, breast, and pancreas (10). This distribution highlights the importance of vitamin D_3_ in many organs. Additionally, VDRs are present in the inner ear, where mutations have been linked to conditions such as sensorineural hearing loss and balance disorders. Research shows that reducing the expression of VDR, particularly its VDRB subtype, results in morphological defects in the inner ear. These defects include malformed semicircular canals and abnormal otolith organs (11). Previous epidemiological studies observed a significant association between vitamin D levels and tinnitus, as well as hearing loss (12).

Due to the limited understanding of the mechanisms behind tinnitus, and previous research suggesting that vitamin D may influence changes in the inner ear, this study aimed to analyze vitamin D_3_ levels and their potential impact on primary subjective tinnitus.

## 2 Materials and methods

### 2.1 Study population

In this study, a total of 350 patients with primary subjective tinnitus and 347 participants serving as a control group were enrolled. The basic parameters of the study populations are presented in Table 1. The inclusion criteria for the tinnitus group required participants to have primary subjective tinnitus, which may include cases associated with sensorineural hearing loss. Additionally, participants needed to provide informed consent to take part in the study, be over 18 years of age, and have all necessary clinical data available. The exclusion criteria included secondary cases of tinnitus and systemic diseases that could affect 25-hydroxyvitamin D_3_ levels. These conditions include malabsorption disorders (such as celiac disease or Crohn’s disease), liver or kidney diseases (like liver cirrhosis or kidney failure), as well as certain medications that may alter vitamin D levels (such as corticosteroids, anti-HIV drugs, laxatives, and cholesterol-lowering medications). Additionally, individuals with obesity (defined as a BMI over 30) or those who have undergone weight loss surgeries were also excluded from the study. All patients received clinical assessments conducted by specialists skilled in tinnitus management. Additionally, all patients underwent audiological evaluations and laboratory tests as outlined below. All participants provided written consent to participate in the study. The investigation adhered to the Declaration of Helsinki and received approval from the Hungarian ETT TUKEB (approval number: BM/29864-1/2024, approval date: December 9, 2024).

**Table 1:** The study populations’ basic parameters.

Parameter	Tinnitus group	Control group	*p*-value
Age (median years; IQR, Q1–Q3)	52 (25; 40–65)	50 (15; 40–60)	0.06*
Sex (men/women)	162/188 (350)	157/190 (347)	0.73**
25-hydroxyvitamin D_3_ levels			
Normal (75 nmol/L<)	163 (46.8%)	237 (68.3%)	<0.00001**
Low (75 nmol>)	187 (53.2%)	110 (31.7%)	
Tinnitus location			
Right, *n* (%)	90 (25.7%)		
Left, *n* (%)	111 (31.7%)		
Bilateral, *n* (%)	149 (42.6%)		
Tinnitus intensity (median dB; IQR, Q1–Q3)	30 (20; 20–40)		
Tinnitus frequency (median Hz; IQR, Q1–Q3)	4,000 (6,000; 2,000–8,000)		
Tinnitus onset (median months; IQR, Q1–Q3)	12 (3–42)		
Hearing loss occurrence			
Sensorineural hearing loss (over 30 dB)	189 (54%)		
Mild (30–40 dB)	90 (48%)		
Moderate (41–60 dB)	78 (41%)		
Severe (61–80 dB)	17 (9%)		
Profound (over 80 dB)	4 (2%)		
Normal hearing levels	161 (46%)		

dB, decibel; Hz, Hertz; IQR, interquartile range; nmol, nanomole; L, liter; Q1, first quartile; Q3, third quartile. *Mann–Whitney *U* test, **Chi squared test. A significance level of *p* < 0.05 was established.

### 2.2 Audiological examinations

Before audiological examinations, all patients underwent microotoscopy, tympanometry, and acoustic reflex testing to rule out potential cases of conductive hearing loss. A qualified audiological assistant conducted pure-tone audiometry and tinnitus matching for each case. These assessments were carried out in a soundproof booth, using headphones for air-conduction testing (ranging from 125 to 8,000 Hz) and a mastoid vibrator for bone-conduction measurements (ranging from 250 to 4,000 Hz). The lowest perceivable intensities were identified in 5 dB increments. The pure-tone audiograms were then constructed manually. Sensorineural hearing loss was defined based on the 1995 recommendations from the Committee on Hearing and Equilibrium of the American Academy of Otolaryngology–Head and Neck Surgery (13). Tinnitus matching started with pitch matching, which aimed to identify the most accurate frequency range of the tinnitus. After this was completed, the intensity of the tinnitus was measured in 1 dB increments at the frequency identified during the intensity matching. The results were then manually recorded on audiograms.

### 2.3 Self-reported severity of tinnitus

Self-reported tinnitus severity was measured using the Tinnitus Handicap Inventory (THI) (14). The THI questionnaire has been previously validated for use with the Hungarian population (15). The THI analyses the impact of tinnitus on daily functioning, using three scales: functional (e.g., social activities, daily activities), emotional (e.g., depression, anxiety), and catastrophic (e.g., loss of control). Patients can answer each question with “yes” (4 points), “sometimes” (2 points), or “no” (0 points). The total THI score is calculated by adding the points from each response. Based on the THI results, tinnitus handicap can be categorized into five levels: no handicap (0–16 points), mild handicap (18–36 points), moderate handicap (38–56 points), severe handicap (58–76 points), and catastrophic handicap (78–100 points). The patients completed the THI questionnaire in the Hungarian language.

### 2.4 Laboratory testing

After a 12-h overnight fast, patients provided venous blood samples after giving their consent. The total serum levels of 25-hydroxyvitamin D were measured using the Atellica^®^ IM Vitamin D Total (VitD) through the Atellica^®^ IM Analyzer (Siemens Healthineers, Siemens Healthcare GmbH, Erlangen, Germany). This measurement employs a competitive immunoassay that utilizes an anti-fluorescein mouse monoclonal antibody covalently attached to paramagnetic particles (PMP), an anti-25-hydroxyvitamin D mouse monoclonal antibody labeled with acridinium ester (AE), and a vitamin D analogue labeled with fluorescein. For the analysis, a 20 μL sample was collected from EDTA-anticoagulated plasma. The specimens were refrigerated at 2–8 °C until analysis was performed. The test’s measuring range is from 10.50 to 375.00 nmol/L. If the 25-hydroxyvitamin D levels exceed 375.00 nmol/L, samples were diluted and retested to ensure accurate results. The assay has a limit of detection (LoD) of 6.83 nmol/L, which is the lowest concentration of vitamin D that can be reliably detected with a 95% probability. Potential interference from hemoglobin, triglycerides, bilirubin, cholesterol, uric acid, human immunoglobulin, and fluorescein has been reported to be 10% or less. The laboratory validated the results, which were also carefully checked by the treating physicians.

In this investigation, serum 25-hydroxyvitamin D levels are categorized into three ranges: low (below 50 nmol/L), insufficient (50–75 nmol/L), and normal (above 75 nmol/L).

### 2.5 Statistical analysis

All statistical analyses were conducted using IBM SPSS version 25 software (IBM Corporation, Armonk, NY, USA). The distribution of the data was assessed using the Shapiro–Wilk test, which revealed a non-normal distribution. As a result, the Mann–Whitney *U* test, a non-parametric test, was utilized. Additionally, categorical analyses were performed using the Chi-squared test. A multinomial logistic regression model was also applied. To analyze sensitivities, receiver operating characteristic curves (ROC) were generated. A significance level of *p* < 0.05 was consistently used throughout the analyses.

## 3 Results

Table 1 presents the basic parameters of the study population.

As shown in Table 1, the control and tinnitus groups did not differ statistically in terms of age (*p* = 0.06) and sex (*p* = 0.73). This confirms the statistical comparability of the two groups. The analysis shows a greater prevalence of females in both groups. Regarding the locations of tinnitus, bilateral and left-sided tinnitus were the most commonly reported. The median duration of 12 months suggests that most cases are chronic tinnitus. Additionally, tinnitus matching results indicate that the tinnitus experienced is generally of higher frequency and more intense. Pure-tone audiometry showed sensorineural hearing loss in 54% of the cohort, with mild (48%) and moderate (41%) forms predominating. The prevalence of low 25-hydroxyvitamin D_3_ levels was significantly (*p* < 0.00001*) higher in the tinnitus group (53.2%) compared to the control group (31.7%).

**Figure 1:**
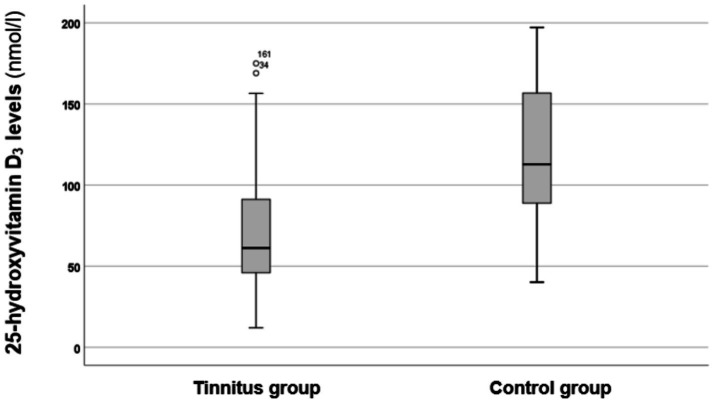
Boxplot illustrating the 25-hydroxyvitamin D_3_ levels in the tinnitus and control groups. The boxes represent the middle 50% of the data, while the whiskers indicate the upper and lower 25%. The black line that divides each box represents the median values. The circles and numbers depict the outliers. Statistical differences were analyzed using the Mann–Whitney *U* test (*p* < 0.05).

In the next phase of the investigation, the 25-hydroxyvitamin D_3_ levels were compared between the tinnitus group and the control group. The results are depicted in Figure 1.

As shown in Figure 1, the tinnitus group exhibited lower levels of 25-hydroxyvitamin D_3_ compared to the control group. A statistical analysis using the Mann–Whitney *U* test indicated that these differences are statistically significant (*p* < 0.00001*, *z*-score: −4.76). Therefore, it can be concluded that individuals in the tinnitus group had significantly lower vitamin D_3_ levels.

In the next step, the effects of 25-hydroxyvitamin D_3_ levels on tinnitus parameters were further evaluated. To accomplish this, a multinomial logistic regression model was implemented.

As Table 2 reports, lower levels of 25-hydroxyvitamin D_3_ significantly predicted the occurrence of tinnitus (*p* = 0.000*; OR: 0.132, 95% CI = 0.066–0.264). Furthermore, vitamin D_3_ insufficiency was a significant predictor of moderately severe and severe tinnitus (*p* = 0.021*; OR: 1.800, 95% CI = 0.806–4.022) as well as the development of chronic tinnitus (*p* = 0.001*; OR: 0.717, 95% CI = 0.384–1.340). However, higher tinnitus intensities (*p* = 0.08; OR: 0.710, 95% CI = 0.302–1.665) and bilateral tinnitus (*p* = 0.549; OR: 1.191, 95% CI = 0.672–2.112) were not significantly predicted by 25-hydroxyvitamin D_3_ levels.

**Table 2:** Multinomial logistic regression model adjusted for age and sex.

Dependent	Predictor	*β*	Std. error	*p-*value		OR	95% CI (lower bound)	95% CI (upper bound)
Tinnitus occurrence	Low 25-hydroxyvitamin D_3_ levels (75 nmol/L>)	−2.023	0.352	0.000*		0.132	0.066	0.264
Moderately severe–severe tinnitus (total THI over 38 points)		0.588	0.410	0.021*		1.800	0.806	4.022
Chronic tinnitus (lasting over 3 months)		−0.725	0.219	0.001*		0.717	0.384	1.340
Higher tinnitus intensity (over 30 dB)		0.534	0.305	0.08		0.710	0.302	1.665
Bilateral tinnitus		0.175	0.292	0.549		1.191	0.672	2.112

A multinomial logistic regression model to analyze the predictors for tinnitus. CI, confidence interval; dB, decibel; nmol, nanomole; L, liter; OR, Odds ratio; Std., standard; THI, Tinnitus Handicap Inventory. The significant results (*p* < 0.05) are indicated with an asterisk (*).

**Figure 2:**
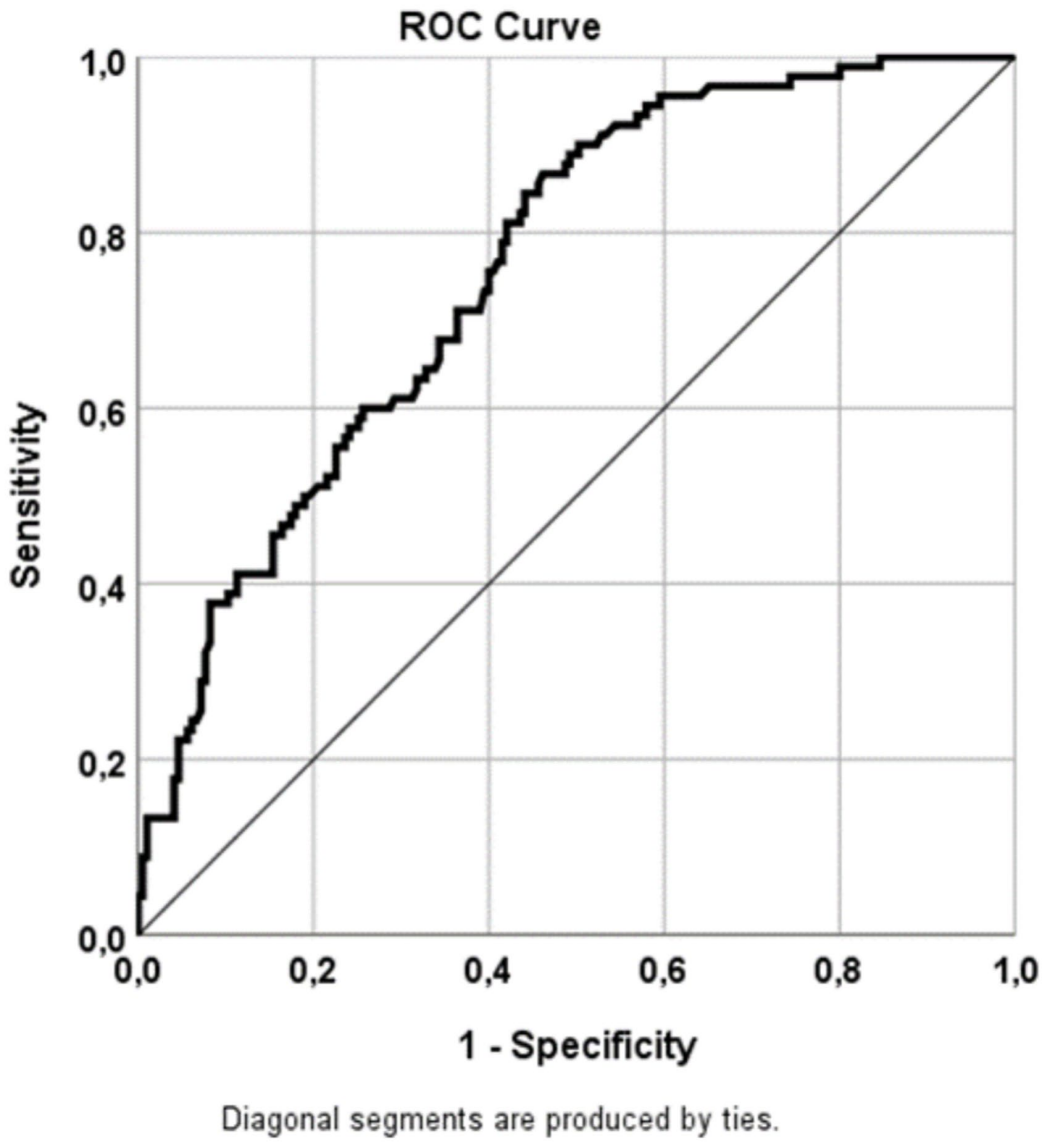
ROC curve illustrating the sensitivities for predicting tinnitus based on 25-hydroxyvitamin D_3_ levels. Sensitivity was calculated from the AUC (refer to Table 3). AUC, area under the curve; ROC, receiver operating characteristics.

In the final step, a ROC curve was generated to analyze the sensitivity of vitamin D_3_ levels in predicting tinnitus (Figure 2).

ROC analysis (Figure 2 and Table 3) revealed that 25-hydroxyvitamin D_3_ levels predicted tinnitus with a sensitivity of 75.8%. This finding was statistically significant (*p* = 0.000*, 95% CI = 0.702–0.815).

**Table 3:** Parameters of the ROC curve analysis.

AUC	Std. error	*p*-value	95% CI (lower bound)	95% CI (upper bound)
0.758	0.029	0.000*	0.702	0.815

AUC, area under the curve; CI, confidence interval; std., standard. The asterisk (*) denotes a statistically significant difference (*p* < 0.05).

## 4 Discussion

This study highlights a significant connection between levels of 25-hydroxyvitamin D_3_ and primary subjective tinnitus. Our main findings reveal that individuals with tinnitus have a notably higher prevalence of vitamin D_3_ insufficiency and significantly lower levels compared to a statistically comparable control group. Additionally, the logistic regression analysis identified low vitamin D_3_ levels as a strong independent predictor for the occurrence, chronicity, and severity of tinnitus.

The existing research on vitamin D_3_ levels in relation to tinnitus is limited. However, our investigation reveals a significantly higher prevalence of insufficient vitamin D_3_ levels among individuals with tinnitus, which is consistent with previous studies. A large-scale study by Aliyeva et al. found a strong inverse correlation between serum levels of 25-hydroxyvitamin D_3_ and the presence of tinnitus (12), suggesting that vitamin D_3_ may play a role in the auditory pathway, potentially influenced by hormonal factors. Other studies have indicated that vitamin D_3_ deficiency may contribute to hearing loss through mechanisms such as cochlear calcium demineralization and impaired microcirculation, leading to morphological changes in the cochlea (16). Investigations have confirmed an additional effect of vitamin D_3_ in the auditory system, highlighting its crucial role in the differentiation and proliferation of neural stem cells and progenitor cells. These cells are essential for maintaining the normal functioning of the auditory nerves (17). Additionally, it has been confirmed that the regulation of the VDR gene through the cGMP-PKG signaling pathway promotes neurite outgrowth and the survival of cochlear spiral ganglion neurons. This further underscores the importance of vitamin D in the cochlear system (18). Our findings strongly support this association. Although the pathophysiological mechanisms connecting vitamin D_3_ deficiency to tinnitus are not yet fully understood, they are likely multifactorial and related to the essential actions of the molecule.

Tinnitus is strongly associated with sensorineural hearing loss (19). Previous studies have shown a negative correlation between vitamin D intake and hearing loss, based on data from the UK Biobank (20). Other studies have also found a link between hearing loss and vitamin D_3_ levels, particularly in populations with diabetes mellitus (21) and among the elderly (22). However, it is important to note that both diabetes mellitus (23) and older age (24) increase the risk of developing sensorineural hearing loss. The current investigation analyzed vitamin D insufficiency in a broader population, including a diverse age range and patients with tinnitus, both with and without hearing loss. As a result, the findings of this study are more generalizable.

At a molecular level, a downregulation of the factors, such as the VDR, due to low vitamin D_3_ could impair auditory processing and increase susceptibility to neural hyperactivity (25). Vitamin D_3_ is a key regulator of neurotrophins like nerve growth factor (NGF) and brain derived neurotrophic factor (BDNF), which are essential for the plasticity and survival of auditory neurons. Consequently, a deficiency can disrupt the transcription of genes critical for cochlear ionic hemostasis, antioxidant defense, and neuroprotection (26). Animal models further highlighted the importance of vitamin D in the development of the auditory system. In particular, in rats with preeclampsia, administering magnesium sulfate (MgSO4) and vitamin D infusions showed a significant role in preventing cochlear degeneration. Scanning electron microscopy revealed that the stereocilia of inner hair cells (IHCs) and outer hair cells (OHCs) exhibited less degeneration across all turns of the cochlea with both vitamin D treatment alone and vitamin D combined with MgSO4 therapy (27). Vitamin D_3_ has notable effects on the endothelium, which is relevant for the inner ear. Research has shown that vitamin D_3_ (but not its precursors) can inhibit the destabilizing effects of inflammatory signals such as interleukin-1β (IL-1β), tumor necrosis factor-*α* (TNF-α), and bacterial lipopolysaccharides. This finding emphasizes the importance of vitamin D in stabilizing the endothelium during inflammation. In addition, a study examining the cerebral arteries of mice on a high-vitamin D diet found that this diet enhanced the leak of a fluorescent reporter through the vessel wall in response to vascular endothelial growth factor (VEGF). These results underscore the critical role of endothelium stabilization in various physiological stimuli (28). This potential effect of vitamin D is significant since endothelial dysfunction seems plausible in inner ear pathologies (29–31).

Vitamin D_3_ not only plays a crucial role in cochlear health at the genomic level, but it also has potent immunomodulatory effects. It reduces the expression of pro-inflammatory cytokines, such as TNF-*α*, IL-1β, and Interleukin-6 (IL-6), while promoting the differentiation of anti-inflammatory T-regulatory lymphocytes (32). Neuroinflammation is increasingly recognized as a significant factor in the development of tinnitus, as it may contribute to glutamatergic excitotoxicity and an increase in central gain within the auditory pathway (33). Given its anti-inflammatory properties, vitamin D_3_ could potentially influence this neuroinflammatory environment, helping to stabilize neural circuits and reduce the central neural plasticity associated with the onset and persistence of tinnitus. Our regression model supports this idea, showing that low levels of vitamin D_3_ are a significant predictor of the development of chronic tinnitus.

Our analysis revealed an association between vitamin D_3_ insufficiency and increased tinnitus severity, as measured by the THI. This suggests that vitamin D_3_ levels may not only influence the onset of tinnitus but also its perceived impact on quality of life. This influence could be mediated by vitamin D_3_’s role in regulating enzymes that are critical for neurotransmitter synthesis, such as tyrosine hydroxylase for catecholamines and tryptophan hydroxylase for serotonin (34). Dysregulation of these pathways is linked to mood disorders, which can significantly affect quality of life. Therefore, a potential connection emerges between vitamin D_3_, emotional well-being, and tinnitus distress. However, it is important to note that our findings did not identify a significant association between vitamin D_3_ levels and increased tinnitus intensity or laterality. This suggests that while vitamin D_3_ may play a role in the pathophysiological mechanisms—through cellular and inflammatory processes—and in the perceived impact of tinnitus, it may not directly influence the peripheral generation of tinnitus or its loudness. This distinction between objective intensity and subjective severity calls for further investigation.

While this study provides compelling evidence, several limitations should be acknowledged. First, the study population was recruited from a clinical setting, which may introduce selection bias by preferentially including individuals with more severe or bothersome tinnitus. Second, the observational design allows the identification of associations but does not permit conclusions regarding causality. It therefore remains unclear whether vitamin D deficiency predisposes individuals to tinnitus, whether tinnitus leads to lifestyle changes that result in vitamin D deficiency, or whether an unmeasured factor influences both conditions. In addition, although major systemic causes of vitamin D_3_ deficiency were excluded, other potentially relevant determinants—such as sun exposure, seasonal variation, and dietary intake—were not assessed. Furthermore, vitamin D deficiency has been linked to mood disorders; however, psychiatric symptoms were not evaluated in this study, representing an additional limitation and an important area for future research. Finally, the lack of measurements for residual inhibition and minimum masking levels further limits the comprehensive characterization of tinnitus-related outcomes. Taken together, these limitations underscore the necessity for well-designed prospective studies and controlled trials to clarify causal relationships and to explore the potential therapeutic effects of vitamin D_3_ supplementation in tinnitus.

## 5 Conclusion

The current investigation provides strong evidence that serum levels of 25-hydroxyvitamin D_3_ are associated with tinnitus. Vitamin D_3_ deficiency has been identified as a significant predictor of the occurrence, chronicity, and severity of tinnitus. While further research is needed to establish a causal relationship, assessing and correcting vitamin D_3_ deficiency may offer a novel, simple, and cost-effective approach to a multidisciplinary management strategy for tinnitus. This could potentially reduce the burden of the condition and improve patients’ quality of life.

## Data Availability

The original contributions presented in the study are included in the article/supplementary material, further inquiries can be directed to the corresponding author.
